# Temporary exclusion of ill children from childcare centres in Switzerland: practice, problems and potential solutions

**DOI:** 10.1186/s12913-018-2831-5

**Published:** 2018-01-15

**Authors:** Benjamin Sticher, Julia Bielicki, Christoph Berger

**Affiliations:** Division of Infectious Diseases and Hospital Epidemiology and Children’s Research Center, University Children’s Hospital Zurich, Steinwiesstrasse 75, 8032 Zürich, Switzerland

**Keywords:** Paediatrics, Infection control, Childcare centres, Standard operating procedures, Temporary exclusion

## Abstract

**Background:**

In childcare centres, temporary exclusion of ill children, if their illness poses a risk of spread of harmful diseases to others, is a central approach to fight disease transmission. However, not all ill children need to be excluded. Previous studies suggested that childcare centre staff have difficulties in deciding whether or not to exclude an ill child, even when official ill-child guidelines are used. We aimed to describe, quantify and analyse these ambiguities and discuss potential solutions.

**Methods:**

For this cross-sectional study, we sent postal surveys to 488 childcare centre directors in the Swiss Canton of Zurich, where no official ill-child guideline is in place. We asked for exclusion criteria for ill children and ambiguities faced when dealing with ill children. We checked whether existing guidelines provided solutions to the ambiguities identified.

**Results:**

249/488 (51%) directors responded to the survey. The most common exclusion criteria were fever (87.4%) and contagiousness (52.2%). Ambiguities were mostly caused by conjunctivitis (23.7%) and use of antipyretic drugs (22.9%). Roughly one third of the ambiguities identified could have been resolved with existing guidelines, another third if existing guidelines contained additional information. For the last third, clear written directives are difficult to formulate.

**Conclusions:**

Written recommendations may help to clarify when an ill child should temporarily be excluded. However, such a guideline should cover the topics antipyretic drugs and teething and have room for modification to local circumstances. Collaboration with a paediatrician may be of additional benefit.

## Background

In Switzerland, Western Europe and the United States overall at least 30%, in several countries up to more than 80% of preschool children enjoy some type of formal care provided in out-of-home childcare centres (CCC) [1–3]. It is well documented that children attending CCCs suffer more infections [4–9] and that CCCs can be the source of and sustain outbreaks of serious infectious diseases [10–12]. As studies have shown decreased transmission rates of infectious diseases in schools during holidays or school closures, temporary exclusion off ill children can – besides review of attendee vaccination status and hygiene precautions – be an important tool in preventing infectious disease transmission in CCCs, too [13, 14]. When deciding whether to temporarily exclude an ill child or not, CCC staff and directors face the challenging task of needing to ensure unaffected children and staff are adequately protected while limiting disruption to the daily routines of the families they serve.

The American Academy of Pediatrics (AAP) in its Guideline for Early Care and Education Programs defines exclusion criteria for ill children in CCC [15, 16]. The presence of highly contagious, potentially harmful infection is one of three key criteria for temporary exclusion. The other two exclusion criteria in the AAP guideline are 1) “illnesses that prevent the child from participating comfortably in activities” and 2) “illnesses that result in a need for care that is greater than the staff can provide without compromising the health and safety of other children” [15]. Being mainly symptom-based and designed for the use by non-medical professionals, endorsed by a nationwide association of medical professionals and being frequently scientifically re-evaluated [17–19] the AAP guideline can be considered as a standard reference for exclusion criteria for ill children in CCC. Due to these properties and its easy access this guideline could be applied more than any other also in Switzerland.

The AAP guideline aims to enable CCC staff to identify ill children that require temporary exclusion. Yet even when applying the guideline, CCC directors are reported to experience difficulties in dealing with ill children. CCC directors tend to exclude children more often than necessary, such as children with mild and harmless illnesses, and frequently do not follow return-to-care recommendations stated in the AAP guideline [17–19]. All of these can negatively impact affected families and may have economic consequences, for example in terms of parental days off work.

Improved support for decision-making in relation to temporary exclusions is desirable. To achieve this, policy makers and practitioners need to better understand CCC directors’ knowledge, attitudes and practices in handling acutely ill children. We asked CCC directors in the Canton of Zurich in Switzerland, a setting without formal guidance, to report their exclusion practice and name ambiguities they experienced. We sought to relate our data to the AAP guideline, to assess the guideline’s applicability and to discuss potential modifications that could better inform CCCs in dealing with decisions about temporary exclusion of ill children.

## Methods

For this cross-sectional study, we designed a paper-based questionnaire for distribution to CCC directors. The questionnaire focused on their handling of ill children, including their self-perception of managing this issue, and eliciting what ambiguities they experience. Data on centre characteristics were also collected. To ensure content validity, questionnaires were piloted and revised by five paediatricians who are in regular contact with CCCs.

Questionnaires were posted to all 488 CCC in the Canton of Zurich (Switzerland). The overall population of Zurich is 1,421,895, of which 5.3% are children less than 5 years of age [20]. Addresses were compiled from a list provided by the local administration office and from entries in a local, Internet based directory [21]. CCC directors who had not responded one month after distribution received up to two telephone reminders. The completion time for the questionnaire was about 30–40 min. In a letter accompanying the questionnaire, CCC directors were informed of the study’s background and goals. They were asked for voluntary participation. In accordance with the institutional review board, the authors did not seek ethics review, as this study did not collect any data on human subjects.

CCC directors were asked in an open-end question to name exclusion criteria for ill children. Additionally, they were asked about the number of children cared for, the number of children less than 2 years of age, the number of staff, whether they had any form of in-house ill-child standard operating procedure (SOP), whether they have an advising paediatrician and if yes, how they collaborate. CCC directors without SOP and/or without an advising paediatrician were asked whether they think that they would benefit from having one. We also asked CCC directors to rate their handling of ill children on a scale from 1 (worst) to 10 (best) and how often they are unsure if an ill child should be excluded (six categories of frequency).

### Ambiguities with the handling of ill children

In order to describe ambiguities with the handling of ill children, CCC directors were asked the following open-ended questions:

1. If you are ever unsure, whether an ill child should be temporarily excluded or not, how do you handle the situation? Please describe.
2. Can you describe situations in which parents did not accept temporary exclusion, although you would have preferred to do so?

Answers to these questions are summarized as ambiguities with the handling of ill children as reported by Swiss CCC directors (Table 3). If a statement could be attributed to several categories, it was exclusively attributed to the category the respondent assumedly wanted to stress. To be listed as a discrete category, an ambiguity had to be mentioned by more than one respondent. For every ambiguity in Table 3, we then checked whether the APP guideline was applicable. If no specific recommendation was provided, we checked whether information was simply lacking in the guideline, or whether it was genuinely difficult to address the issue through written guidance. We referred to the 2011 version of the AAP guideline, which is still consistent with the updated 2015 version concerning the topics discussed in this study.

### Data analysis

Descriptive analyses were undertaken using IBM SPSS Statistics version 21 (SPSS Inc., Chicago, IL). Differences between means were assessed using the independent two-tailed t-test. *P*-values smaller than 0.05 were considered significant.

## Results

### Characteristics

In total, 249/488 CCCs responded to the survey (51,0% response rate). The response rate was lower in its urban centre (Zurich city; 43.1%) than in the rest of the Canton (58.2%). On average, participating CCCs had 10.1 full time equivalent staff caring for 26 children (range 4–78) with a total number of 6424 children being served by the surveyed CCCs. 88.0% of the CCCs accepted children less than 2 years of age. Only 7.6% of the responding centres were part of a multi-centre childcare organization. Both private and public CCCs participated.

### In-house guidelines and policy

The majority of centres (85%) reported having in-house ill-child standard operating procedures (SOP). 53% of those without SOPs said they would benefit from such documentation. 73% of all respondents reported having a named advising paediatrician. The level of this collaboration differed widely, from simply designating a responsible paediatrician in order to fulfil local administrative requirements to intensive and on-going cooperation including joint drafting of in-house ill-child SOPs, on-site visits and provision of telephone advice. 41% of the CCCs without a named paediatrician stated they would benefit from such collaboration.

### Exclusion criteria for ill children

Tables 1 and 2 summarize the circumstances and symptoms that reportedly lead to temporary exclusion of ill children. In addition, specific diseases mentioned as reasons for temporary exclusion from CCCs included gastroenteritis (20.9%), head lice (9.6%), chickenpox (8.8%), flu-like disease (6.4%), measles (6.0%).

**Table 1:** Exclusion criteria for ill children as reported by Swiss CCC directors: Medical and social circumstances

Category	%^a^	Exclusion criterion	%
Contagiousness	52.2	Not specified	47.4
		If contagiousness is suspected; until a physician confirms that there is no threat	1.6
		Except for common colds	1.6
Extent of Illness	45.3	Discomfort, pain or altered general condition	29.7
		Any sign of illness	7.2
		If the child cannot participate comfortably in daily activities	6.4
		Any sign of illness in the last twenty-four hours	2.0
Institutional limits	8.4	If the child needs medication	2.8
		If we cannot provide optimal care	2.4
		If the child needs the care of its parents	2.0
		If the child needs to see a physician	1.2
Interference with other children’s need	3.2	If the child needs more attention than we can offer	2.4
		If not being excluded would be possibly dangerous for the child or the other children	0.8

^a^This number includes once-only mentions, which are not further described in this table

**Table 2:** Exclusion criteria for ill children as reported by Swiss CCC directors: Symptoms and signs

Category	%^a^	Exclusion criterion	%
Fever	87.4	Not specified	36.1
		Above a defined body temperature (≥ 38, 38.1 or 38.5 °C)	35.3
		High fever	8.0
		With other signs of illness	3.6
		If it is not due to teething	1.2
Conjunctivitis	29.3	Not specified	20.1
		Eye irritations, e.g. tearing or red eyes	4.4
		Until a physician confirms that there is no threat	1.6
		Until a defined time after beginning of treatment	1.2
Vomiting	19.7	Not specified	16.9
		More than once	1.6
		Repeated vomiting	0.8
Diarrhoea	18.0	Not specified	10.4
		Above a defined number of unformed stools	3.6
		Heavy diarrhoea	2.8
Various symptoms	9.6	Abnormal breathing	2.8
		Rash of unclear origin	2.8

^a^This number includes once-only mentions, which are not further described in this table

### Ambiguities in decision-making about temporary exclusion

Table 3 shows situations in which CCC directors experienced ambiguity about temporary exclusion of affected children. The AAP guideline covers 39% of these, most often when CCC directors are unsure about the handling of conjunctivitis, rashes and fever. The remaining 61% of described situations can be divided into two groups: (i) those for which an SOP could provide a standardized solution, but for which information is lacking in the AAP guideline (listed as IL in Table 3); (ii) those, which are difficult to address in a written directive.

(i) Ambiguous situations that could be addressed through an SOP.

**Table 3:** Ambiguities with the handling of ill children as reported by Swiss CCC directors

Description of the ambiguous situation	%^a^	AAP?^b^
Conjunctivitis	23.7	
Not specified	8.4	Yes
Unclear when or for how long conjunctivitis is contagious	5.6	No; MK
Differentiation between infectious and non-infectious conjunctivitis	4.4	Yes
Parents want to bring the child without seeing a doctor first	1.6	Yes
Parents insist on conjunctivitis not being contagious	1.6	No; PC
Different doctors give different recommendations	1.2	No; CWP
Antipyretic drugs	22.9	
If a feverish child has received antipyretic drugs and is increasingly ill, as the effect is fading	13.3	No; IL
Parents administer antipyretic drugs without informing us	12.4	No; IL
Not specified	3.2	No; IL
Parents insist on inclusion after use of antipyretic drugs	2.8	No; IL
Parents want us to administer antipyretic drugs	0.8	No; IL
Rashes	16.5	
We cannot gauge the cause	6.0	No; MK
Not specified	3.6	Yes
Chickenpox; parents want to bring children before all lesions are dry	3.2	Yes
Hand foot and mouth disease	1.6	Yes
Teething	10.4	
We are not sure, if teething causes illness, diarrhoea or fever	5.2	No; IL
Parents insist on teething causing illness, diarrhoea or fever	3.2	No; IL
Not specified	2.0	No; IL
Fever	10.0	
If the child is ill, but does not have fever; or vice versa	5.6	Yes
If the child had fever in the past 24 h, but is well now	3.6	Yes
Not specified	1.6	Yes
Vomiting	5.6	
Parents insist on inclusion or find excuses like eating too much	2.4	No; PC
Vomiting only once	1.6	Yes
Not specified	0.8	Yes
Diarrhoea	5.2	
Parents insist on inclusion or do not think it is diarrhoea	2.0	No; PC
Not specified	1.2	Yes
Child has diarrhoea, but is normally active	0.8	Yes
Only one unformed stool	0.8	Yes
Other	24.9	
If exclusion criteria are met, but there is no alternative care available	5.2	No; OAC
Common cold	4.8	Yes
Parents give us wrong or incomplete information	3.6	No; PC
Child is uncomfortable without any obvious reason	2.8	Yes
Parents do not have the same perception of when a child is ill	2.8	No; PC
If we think a child should be excluded, but the paediatrician does not	2.4	No, CWP
Parents call us in the morning to ask whether we care for their ill child	1.2	No; PC
If one of our attendees’ family members has an infectious disease	1.2	Yes
Lice	1.2	Yes
Oral infections	0.8	Yes
Different doctors give different recommendations	0.8	No; CWP
Suspicion of contagious diseases	0.8	No; MK

This table integrates answers to survey questions 1) and 2), as mentioned in Methods

^a^the sum of the percentages of the subcategories may not be equal to the total percentage of the category, due to (i) once-only mentions not described in the table but counted for the category’s total percentage and (ii) directors who stated several ambiguities (subcategories) within one category

^b^Procedure provided by the AAP guideline? If not: What is the issue underlying this ambiguity? Possible answers, if the AAP guideline does not state a procedure: Information lacking in the current AAP guideline (IL), parental communication (PC), medical knowledge (MK), organization of alternative care (OAC), collaboration with paediatricians (CWP)

There are two commonly named situations that could be managed according to an SOP such as the AAP guideline, but for which information is lacking in the current AAP guideline:

#### Antipyretic drugs

A majority of the 23% of respondents who named antipyretic drugs as a source of ambiguities indicated that a particularly problematic situation was when they suspected parents of giving their children antipyretic drugs in the morning to avoid exclusion for fever or illness. More than half of these respondents added that parents would often fail to inform staff about antipyretic use and/or elevated temperature observed at home. Staff stressed that this lack of information was particularly problematic when the drug effect was fading, and they were confronted with deterioration in the child’s general state.

#### Teething

Some respondents reported that they were not able to determine whether certain symptoms, such as fever, were related to teething (5.2%). Further, 3.2% reported that parents would insist on inclusion of their ill child if they believed that symptoms could be explained by teething.

(ii) Ambiguous situations not easily addressed through an SOP.

This second group can be subdivided into four basic issues: Parental communication, medical knowledge, organization of alternative care and collaboration with paediatricians.

#### Parental communication

13.6% of the respondents mentioned difficulties in decision-making on temporary exclusion related to insufficient or difficult communication with parents. Most frequently, parents were reported to insist on the inclusion of a child with a potentially contagious disease. CCC directors also reported having disagreements with parents regarding the significance of their child’s symptoms for the child or for other children: “Parents start discussing about the definition of diarrhoea without understanding that this is contagious for other children” (Quote from survey).

#### Medical knowledge

Their lack of medical knowledge was a major challenge for many directors (12.6%). Those naming conjunctivitis as a potential reason for temporary exclusion, for example, reported uncertainty in differentiating between infectious and non-infectious conjunctivitis, and regarding the contagiousness of the disease. Some 6% reported not to be able to gauge the cause of a rash, and therefore being unsure of how to handle the situation.

#### Organization of alternative care

5.2% of the respondents reported problems when excluding an ill child, if no alternative care is available. This is most common when parents are not able to leave their workplace to look after their ill child themselves.

#### Collaboration with paediatricians

4.4% of the respondents specifically expressed that some situations were challenging because of a lack of structured paediatric support. The most common complaint was that different doctors contacted ad hoc when the need arose gave different recommendations, for example with regard to the management of conjunctivitis.

### Directors’ self-assessment

**Fig. 1:**
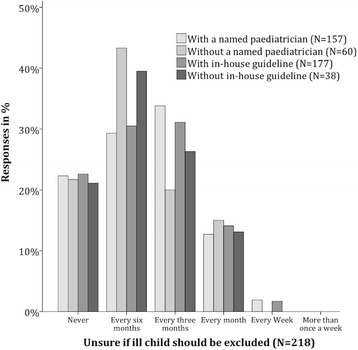
“How often are you unsure, whether an ill child should be temporarily excluded or not?” (*N* = 218)

Asked to rate their handling of ill children on a scale from 1 (worst) to 10 (best), 87% chose a grade from 7 to 9, the overall average being 7.97. The directors’ rating of their handling of ill children was higher, but not significantly, in institutions with a named paediatrician compared to institutions without a named paediatrician (8.01 vs. 7.83, *p* = 0.373). This also applies to institutions with in-house ill-child SOPs compared to institution without in-house ill-child SOPs (8.03 vs. 7.61, *p* = 0.103)**.** The frequency of being unsure whether or not to exclude an ill child did not differ significantly either between institutions with and without a named paediatrician, or between institutions with and without in-house ill-child SOPs (Fig. 1).

## Discussion

Our analysis of the handling of ill children in 249 Zurich-based childcare centres (CCCs) and of ambiguous situations reported by CCC staff in relation to temporary exclusion of ill children identified fever and contagiousness as the two most common exclusion criteria, and conjunctivitis and antipyretic use as the most commonly reported ambiguous situations.

Participating CCC directors had a positive self-perception of their handling of ill children. Nevertheless, providing continuous care, respecting the needs and health of all children at the centre, avoiding the spread of dangerous infectious diseases and ensuring economic profitability were reported to potentially compete with each other and could pose challenges in decision-making about temporary exclusion of ill children. Conjunctivitis, antipyretic drugs, rashes, teething, fever, vomiting and diarrhoea were, in this order, most frequently mentioned as being potentially challenging (Table 3). We hypothesize that some of these situations were already addressed by available guidance, that others could be addressed by written SOPs, and that further still a named CCC paediatrician could help in evaluating situations that cannot easily be addressed by a written directive.

In Switzerland, where this study was conducted, there is no official guideline for CCCs similar to the American Academy of Pediatrics (AAP) Guideline for Early Care and Education Programs [15, 16]. The AAP guideline could provide support to better manage 39% of all reported ambiguous situations (Table 3). Additionally, we identified two situations frequently mentioned by Swiss CCC directors that are not specifically addressed by the AAP guideline: teething and interpretation of symptoms in the context of antipyretic drugs. Written recommendations endorsed by professional bodies, like the AAP, for these issues would be helpful.

Even though many people, including health care professionals [22], believe that fever or diarrhoea may be caused by teething, evidence supporting this is lacking [22, 23]. Recent studies point to possible confounders like waning of maternal antibodies or exposure to a wide variety of childhood illnesses that both occur at the same age as teething [23]. Therefore, a statement to this effect might clarify existing guidance, in particular in conjunction with the statement that “teething should not influence decision on temporary exclusion from CCC”.

The use of over-the-counter antipyretic drugs can improve the overall comfort and wellbeing of a child [24], and allow it to participate in CCC activities. However, their use can also be challenging for the CCCs. Parental administration of antipyretics in the morning temporarily lowers fever and improves wellbeing, but has no effect on the underlying condition. When the drug effect fades, it may be necessary to administer further doses. This requires a number of conditions to be fulfilled, including but not limited to staff training and logistic capacity [15]. Regardless of whether they fulfil these conditions or not, some CCCs may prefer febrile children to be primarily comforted by their parents. Whether CCCs support the use of antipyretics or not, it is crucial to encourage and maintain an atmosphere of open communication between parents and CCC staff to prevent misinterpretation of the child’s general state and behaviour resulting from concealed administration of antipyretics at home. A statement to the following effect may provide clarification for all concerned: The CCC define in their policy and communicate to parents if they are willing and able to provide care for children requiring fever-lowering medication. As the effect of such medication is often temporary, this should be offered only if CCC staffs are trained and happy to administer fever-lowering medication and if parents are able and willing to pick up their children if their condition deteriorates.

In addition to the situations covered by the AAP guideline and recommendations for teething and antipyretic drugs that could be added as discussed above, some ambiguities are likely to persist. The most pressing of these less well-defined issues are likely to be CCC-specific, and may be related to the administrative structures, such as the population and number of children for whom care is provided, staffing levels and other similar factors. We identified four basic unifying themes for these types of ambiguous situations: parental communication, medical knowledge, organization of alternative care and collaboration with paediatricians. We propose a productive and structured collaboration with a named paediatrician or other adequately trained healthcare professional as essential to deal with the majority of these basic issues.

In our study, directors who have a named paediatrician did not rate their performance significantly higher than directors without a named paediatrician. However, previous studies demonstrate that the presence of childcare health consultants improves health-related childcare quality [25, 26]. Directors seem to profit from experiencing a greater consistency of medical advice, because they have one go-to medical professional contact. Such professional contacts may help CCCs clarify repeated ambiguities by developing CCC-specific SOPs, help staff anticipate challenging situations and provide advise for less clear-cut situations. Thus collaboration of a CCC with a paediatrician might contribute to clearer exclusion criteria and an improved handling of ill children in general.

Consistency in the handling of ill children may facilitate the communication with parents and ease the atmosphere in and around the CCC. This is likely to be key with respect to communication with parents as a source of ambiguities. Naturally, the parents’ focus tends to be on their child and family unit, and they may underestimate the needs of other children or institutional limitations. If as a result staff and parental views on handling acute illness diverge, a named healthcare professional could act as a neutral, professional third party, representing both individual child and public health needs. Furthermore, healthcare professionals could educate staff about health-related topics and thereby close gaps in medical knowledge. This includes issues beyond the management of specific situations, such as conjunctivitis. For example, enhancing broader staff understanding of the meaning and consequences of fever could be useful, as more than 80% of the respondents mentioned fever per se as an exclusion criterion (Table 2). Moreover, staff should be educated about the differentiation between diseases that are contagious and diseases that are both contagious and dangerous. At the moment, almost every second respondent listed contagiousness as an exclusion criterion, even though this may not always be medically indicated. If the risk for severe disease or outbreaks do not outweigh the costs of exclusion, as for example in the case of simple running nose, contagiousness as such is not an adequate exclusion criterion.

### Limitations and strengths

There are three potential limitations to our study. First, our study population may not be representative for other regions in Switzerland and elsewhere. Second, we cannot exclude a social desirability bias. Respondents might have underemphasized specific ambiguities with the handling of ill children, as questions were open-ended. Third, as participation was voluntary, we have to assume a non-response bias of unclear direction. The reasons for the lower response rate in the urban centre of the region covered in this study remain unclear. Generally, it is possible that CCCs with more professional handling of ill children were more likely to participate in the survey. Nevertheless, our study provides important insight into reported handling of acutely ill children at Swiss CCCs. To our knowledge, no previous study has provided CCC directors with the opportunity to give detailed feedback on difficulties they experience when they are temporarily excluding ill children. By asking respondents to tell us about these situations and describe them in their own words, widely experienced challenges that have not been described previously were identified.

## Conclusion

In regions where currently official guidance on handling acute illness of children at CCCs is lacking, written recommendations based on the AAP Guideline for Early Care and Education Programs might help CCC staff in deciding about temporary exclusion of ill children. Fever and contagiousness, being rather non-specific complaints, are the most frequently mentioned clinical syndromes for temporary exclusion in Swiss CCCs. Application of the AAP guideline would help to improve indiscriminate current practice and provide procedures to problematic situations. However, the AAP guideline has important gaps that would need to be addressed for it to be applicable in the Swiss context. Besides conjunctivitis, ambiguities experienced by CCC staff commonly related to the use of antipyretic drugs. To address these and other ambiguous situations, the AAP guideline could be complemented by SOPs for individual needs and a close collaboration with a named paediatrician. This would contribute to clear, open and constructive communication between staff and parents, and could lead to more evidence-based, less controversial management of children with fever, self-limiting infections or other currently inadequately handled situations. This may result in a safe reduction of unnecessary and controversial exclusions from CCC, which would be in the interest of families and staff.

## Abbreviations

AAP: American Academy of Pediatrics
CCC: Childcare centres
SOP: Standard operating procedures
